# Conjunctivitis and Total IgE in Lacrimal Fluid: Lacrytest Screening

**DOI:** 10.1155/2009/518903

**Published:** 2009-04-12

**Authors:** Susana Monzón, Elena Arrondo, Joan Bartra, Ferran Torres, María Basagaña, M. del Mar San Miguel, Rosario Alonso, Anna Cisteró-Bahima

**Affiliations:** ^1^Allergy Department, Dexeus Institute, Universitat Autònoma de Barcelona (UAB), 08028 Barcelona, Spain; ^2^Ophthalmology Department, Mutua de Terrassa Hospital, 08221 Barcelona, Spain; ^3^Laboratori de Bioestadística i Epidemiología (LBE), Universitat Autònoma de Barcelona (UAB), 08193 Barcelona, Spain; ^4^Servei de Farmacologia Clínica, Unitat d'Avaluació Prevenció i Suport (UASP), Hospital Clínic, 08036 Barcelona, Spain

## Abstract

Total tear IgE has been considered to play an important role in allergic conjunctivitis, and measurement has been considered useful for diagnosis. The aim of this study was to ascertain whether Lacrytest^®^, a new commercialised method to detect IgE levels in lacrimal fluid, could constitute a screening test for the diagnosis of allergic conjunctivitis. This was a cross-sectional study. Patients with seasonal and perennial allergic conjunctivitis, vernal keratoconjunctivitis and a control group were included. Clinical history, ophthalmic examination, skin prick test and conjunctival provocation test were obtained. Lacrytest^®^ was later performed in all groups. Fifty-four patients were enrolled: thirty with IgE-mediated conjunctivitis and, nine with vernal keratoconjunctivitis and fifteen controls. Lacrytest^®^ was negative in all controls, positive in 20% of the IgE-mediated conjunctivitis group and in 88.9% of the vernal keratoconjunctivitis group. Global statistically-significant differences were found among the three groups (*P* = .003). Sensitivity of the test in the IgE-mediated conjunctivitis group was 20%, specificity 100%, positive predictive value 100%, and negative predictive value 38.46%, while in VKC sensitivity was 88.88%, specificity 100%, positive predictive value 100%, and negative predictive value 93.75%. Our data confirm that this test is not useful for screening allergic conjunctivitis. Lacrytest^®^, while not providing any useful information to an allergist, could be helpful for ophthalmologists to confirm an IgE-mediated or VKC conjunctivitis.

## 1. Introduction

Allergic conjunctivitis constitutes a group of diseases affecting the ocular surface; however, different kinds of conjunctival disorders are grouped under this umbrella term for this single clinical entity. Seasonal and perennial allergic conjunctivitis (SAC and PAC) can be defined as recurrent and bilateral conjunctival inflammation with exacerbations in different seasons of the year caused by direct exposure of the ocular surface to airborne allergens. Both are mainly dependent on classical type I hypersensitivity in which patients have positive skin prick tests and specific IgE in serum to airborne allergens. Itching is the major symptom in this type of conjunctivitis. Ocular findings are scant or even absent and are not related to symptom intensity [1].

Vernal keratoconjunctivitis, a chronic severe inflammatory disease of the conjunctiva usually recurring bilaterally and seasonally (spring and summer), occurs predominantly in male children and young adults with a personal or family history of atopy. Itching is the most significant symptom in these patients, although cobblestone papillae, excess mucus, and intense photophobia may be observed. Corneal involvement may occur and result in permanent vision damage. The pathogenesis is more complex than that of SAC and PAC, and a leading role of an inflammatory network not confined to the classical IgE-mast cell immediate hypersensitivity paradigm, but characterised mainly by Th2-type inflammation with mast cells, basophils, eosinophils, and polyclonal IgE activation, has been suggested. SPT and serum specific IgE antibody test are often not positive, although total serum IgE levels are high. Eosinophils are present in both tears and conjunctival scrapings [2].

A new lacrimal test based on total IgE determination has been commercialised to diagnose allergic conjunctivitis. Total tear IgE has been considered to play an important role in allergic conjunctivitis and it has been shown that the measurement of tear IgE concentrations can aid the diagnosis of this condition [3–5]. Lacrytest (ADIATEC S.A, Nantes, France) is a rapid immunoassay for total IgE determination in tears. This assay indicates, in a qualitative manner, the presence of total class E immunoglobulin in tears with levels above the normal value (<2 KU/L, 3 ng/mL) [3]. In order to investigate whether Lacrytest could be a screening tool to diagnose allergic conjunctivitis, we analysed the results of the test in patients with allergic conjunctivitis and compared them with a control group in a cross-sectional study.

## 2. Methods

### 2.1. Patients and Study Design

Patients were systematically enrolled from October 2004 to April 2005. The study included two centres: Institute Universitari Dexeus of Barcelona (Allergy Department) and Mutua of Terrassa (Ophthalmology Department). Patients were preselected according to a clear history of allergic conjunctivitis. A clinical history was taken and an ophthalmic examination and finally a skin prick test (SPT) to airborne allergens and a conjunctival allergen provocation test (CPT) were performed if the SPT was positive. Antihistamines were prohibited for three days before skin testing and conjunctival challenge. Selected patients gave their written informed consent.

Patients were divided into three groups depending on their diagnosis. The vernal keratoconjunctivitis (VKC) group was based on clinical history and ophthalmic examination (giant papillae or superficial keratitis). SPTs were not considered because are often not positive [2].

Seasonal and perennial allergic conjunctivitis (IgE-mediated allergic conjunctivitis) were diagnosed by clinical history, positive SPT to pneumoallergens and a positive conjunctival-specific challenge test. Ophthalmic examination was not a basis to diagnose them because they are acute forms of conjunctivitis and some patients could not have ocular symptoms and signs of active allergic conjunctivitis at the moment of the visit.

The control group comprised patients with no symptoms of allergy (atopic dermatitis, rhinitis, or asthma) or conjunctivitis in their clinical history, and with normal ophthalmic examination and negative SPT.

After the division into three groups and with or without signs of active allergic conjunctivitis in that moment, Lacrytest was performed in one eye for the control and vernal keratoconjunctivitis groups and in both eyes for the IgE-mediated allergic conjunctivitis group: in one eye immediately after the conjunctival-specific challenge test and in the other in which the allergen was not instilled to compare the results between eyes.

### 2.2. Skin Prick Test

All patients underwent SPT performed according to standard procedure [6] with *Dermatophagoides pteronyssinus*, *Dermatophagoides farinae*, *Dermatophagoides microceras,* cat and dog epithelium, moulds (*Aspergillus fumigatus, Alternaria alternata*), pollens (*Phleum pratense*, *Cynodon dactilon*, *Phragmites comunis*, *Olea europaea*, *Platanus acerifolia*, *Cupressus arizonica, Pinus radiata, Parietaria judaica*, *Artemisia vulgaris, Chenopodium album*, *Plantago lanceolata)*, latex, and cockroach (*Blatella germanica*). These allergens were supplied by ALK-ABELLÓ (Madrid, Spain). Histamine and isotonic saline were used as positive and negative controls, respectively. Mean wheal diameters of 3 mm were considered a positive reaction.

### 2.3. Conjunctival Allergen-Specific Provocation Test (CPT)

This test was performed with the allergen suspected of being one of the aetiologic causes of IgE-mediated conjunctivitis in each patient, chosen on the basis of clinical history correlation and SPT results. The CPT was conducted according to Möller et al. [7]. Each patient was skin prick tested on the volar surface of the forearm with four ten-fold serial dilutions of the specific allergen extract; the CPT was started with the dilution before the one that was positive in the SPT (mean wheal diameter 3 mm) and one drop was instilled into the conjunctival sac. The dilutions instilled were increased every twenty minutes until the test proved positive. Criteria for a positive test were congestion of the conjunctival mucosa, itching, and eye watering.

### 2.4. Lacrytest

Lacrytest is an assay for total IgE determination in tears. A strip is placed in the lower conjunctival fornix and, when wet with tears, is removed. Total IgE reacts with a gold-labelled antibody and is immobilised with the uptake of anti-IgE antibody. Signal intensity is dependent on the total IgE level. For normal value, below 2.5 KU/L, no line was obtained. The positive results could be divided among the three groups on a semiqualitative scale: for low total IgE levels, 2.5 to 10 KU/L, intensity of the signal in the IgE reactive field was lower than that of the control line; for medium total IgE levels, 10 to 40 KU/L, intensity was closer to that of the control line; and for high total IgE levels >40 KU/L, intensity was stronger than the control. The test was performed according to the manufacturer's instructions.

### 2.5. Statistical Analysis

A descriptive analysis of demographic characteristics and clinical symptoms was carried out. The following statistical tests were used for the inferential analysis: Fisher's exact test for qualitative variables, Mann-Whitney U or Kruskall-Wallis tests (for 2 or 3 groups, resp.) for semiquantitative ordinal measurements, and the MacNemar test for binary paired data. Sensitivity, specificity, and positive predictive value were calculated with standard formulae [8]. The analysis was performed using SPSS v10.0 *(SPSS, Inc., Chicago IL)*, and the CIA software for calculation of the 95% confidence intervals [9]. The level of significance was established at *P* = .05 (two-sided).

## 3. Results

Fifty-four patients (30 males, 24 females) were included. Mean (standard deviation) age was 30.6 (20.2) years (range: 5–76). The main demographic characteristics and different clinical conjunctivitis groups are summarised in Table 1.

**Figure 1:**
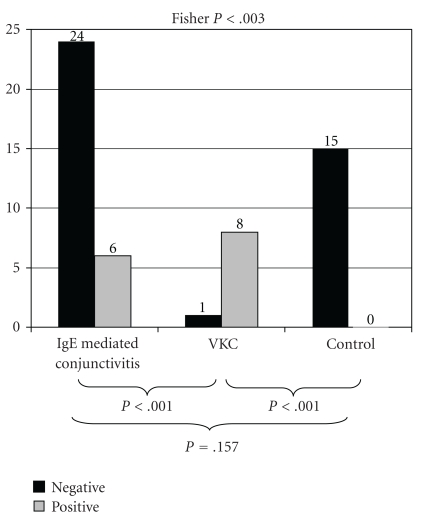
Lacrytest global results. Number of subjects is shown on the top of each column.

The Lacrytest was positive in only 6 patients with IgE-mediated conjunctivitis (20%) and 8 patients with vernal keratoconjunctivitis (88.9%) and was negative in all controls (100%). Differences were significant when the three groups were compared (*P* = .003); pair-wise comparisons of VKC versus the other groups were statistically significant (*P* < .001 for both comparisons), but not between IgE-mediated conjunctivitis and control groups (*P* = .157). (Figure 1).

**Figure 2:**
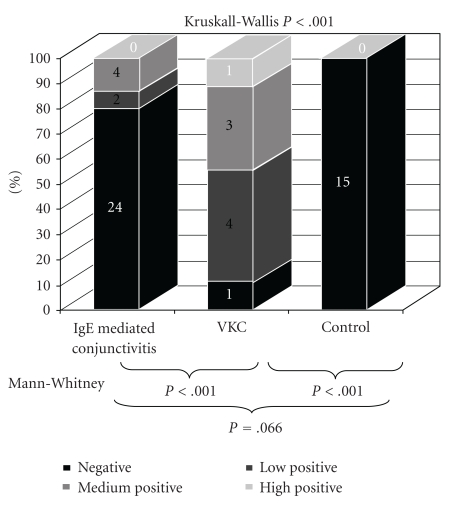
Lacrytest qualitative results. Number of subjects is shown on the top of each column.

If the result of the ocular test in its positive semiqualitative scale (negative, low, medium, and high) is analysed, the results among the three groups were also significant (*P* < .001); pair-wise comparisons of control versus VKC and VKC versus IgE-mediated conjunctivitis were statistically significant (*P* < .001 for both comparisons), but not between control versus IgE-mediated conjunctivitis groups (*P* = .066) (Figure 2).

**Figure 3:**
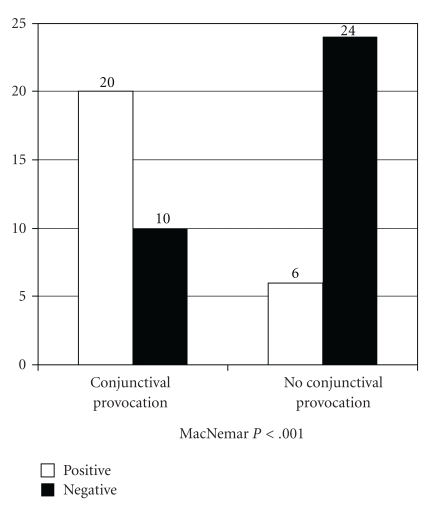
Lacrytest results in IgE-mediated conjunctivitis group.

Results of the lacrimal test used in IgE-mediated conjunctivitis improved after the conjunctival provocation test had been applied to the contralateral eye in each subject. All 6 previously positive results remained positive, but 14 new positives were observed among the 24 previously negative (58.3%, McNemar test *P* < .001) (Figure 3).

Regarding the Lacrytest results (Table 2), in the IgE-mediated conjunctivitis group sensitivity was 20%, specificity 100%, positive predictive value 100%, and negative predictive value 38.46%, while in VKC sensitivity was 88.88%, specificity 100%, positive predictive value 100%, and negative predictive value 93.75% (Table 3). If results after the CPT were analysed in the IgE-mediated conjunctivitis group (Table 2), sensitivity was 66.66%, specificity 100%, positive predictive value 100%, and negative predictive value 60%.

## 4. Discussion

The eye is one of the major targets of allergic disorders, either alone or accompanied by other allergic affection [10]. The ocular component may be the most prominent and sometimes disabling feature of the allergy. The acute forms (seasonal and perennial allergic conjunctivitis) are commonly seen in an Allergy department, and over 50% of patients also have a history of rhinitis, whereas the more chronic forms [11] (vernal keratoconjunctivitis, atopic keratoconjunctivitis, and giant papillary conjunctivitis) can be seen more often in an Ophthalmology department since patients report only ocular symptoms. The acute forms are mediated by a type I hypersensitivity response and the chronic forms have a more complex immunological basis and a chronic inflammatory component [2].

Based on the finding of high total IgE levels in allergic conjunctivitis tears, a new diagnostic method has been commercialised (Lacrytest. ADIATEC S.A., Nantes, France), which qualitatively indicates the presence of total class E immunoglobulins in tears with levels above normal values. Positive levels of the test indicate an allergic conjunctivitis and negative levels rule out this aetiology.

We decided to evaluate the lacrimal test because it was rapid and easy to perform and a prospective study was designed to ascertain its reliability. Our clinical data showed the Lacrytest to be positive in only 6 of 30 patients (20%) with IgE-mediated conjunctival allergy, confirmed by SPT and CPT. This is a very low value compared with other studies in which the IgE-mediated conjunctivitis group showed the most pronounced local IgE production [12, 13]; however the test showed an increased positive value after conjunctival specific provocation (20 of 30 patients; 66.7%). Tear IgE levels are known to have a significant influence on intensity of the inflammatory process and the correlation between CPT and lacrimal IgE has been demonstrated [14]. During allergic reactions, the local synthesis of IgE is elevated and total tear IgE increases because of the lacrimal IgE that have filtered through the blood-tear barrier [15]; thus, it is logical that the results proved more positive after exposure to the allergen. In any event, these findings merit further studies to confirm and explain this result.

In the vernal keratoconjunctivitis group, the Lacrytest was positive in 8 of 9 patients (88.9%). The results obtained in VKC concur with those in the literature. Locally-produced IgE levels have been shown to be the largest contributor to the severity of allergic conjunctivitis [16], particularly in chronic forms, and local IgE production in VKC is increased compared with controls [13, 14] although no clinical differences were observed in atopic keratoconjunctivitis (AKC) [17].

The Lacrytest has very high specificity and positive predictive value (100%); thus, a positive test could point to an allergic aetiology, but the low number of positive cases found in the group of IgE-mediated allergy (sensitivity 20%, negative predictive value 38.46%) indicates that this test cannot be the only parameter to achieve an aetiological diagnosis, as occurs with serum IgE. This test is not useful for allergists because we have other more sensitive and specific methods to diagnose IgE-mediated allergic conjunctivitis; however, it could be helpful to ophthalmologists to confirm an IgE-mediated reaction or VKC if the result is positive. Negative results should be sent to the allergist for the allergic aetiology to be definitively confirmed or ruled out.

## Figures and Tables

**Table 1:** Demographic characteristics and groups of study.

Patient	Group	Sex	Age	SPT	Patient	Group	Sex	Age	SPT
1	1	F	14	+	31	2	M	8	−
2	1	M	37	+	32	2	M	5	−
3	1	F	52	+	33	2	M	65	−
4	1	F	12	+	34	2	M	9	−
5	1	F	39	+	35	2	F	25	−
6	1	F	28	+	36	2	F	76	−
7	1	F	49	+	37	2	F	74	−
8	1	M	30	+	38	2	F	74	−
9	1	F	24	+	39	2	F	14	−
10	1	M	21	+	40	2	M	34	−
11	1	F	25	+	41	2	F	52	−
12	1	F	14	+	42	2	F	43	−
13	1	F	15	+	43	2	F	36	−
14	1	F	43	+	44	2	M	25	−
15	1	M	33	+	45	2	F	17	−
16	1	M	24	+	46	3	F	8	−
17	1	F	39	+	47	3	M	9	−
18	1	M	33	+	48	3	M	10	+
19	1	M	36	+	49	3	F	7	+
20	1	M	28	+	50	3	F	6	+
21	1	M	27	+	51	3	F	8	−
22	1	F	33	+	52	3	M	9	−
23	1	F	18	+	53	3	F	9	−
24	1	F	70	+	54	3	M	8	−
25	1	M	55	+					
26	1	M	13	+					
27	1	F	59	+					
28	1	M	56	+					
29	1	M	39	+					

30	1	M	56	+					

1- IgE-mediated conjunctivitis group. 2- Control group. 3- VKC. M: male. F: female. +: Positive SPT. −: Negative SPT.

**Table 2:** Sensitivity and specificity of the Lacrytest (IgE-mediated conjunctivitis before and after CPT *versus* control group).

	IgE-mediated conjunctivitis before CPT	Control	Total		IgE-mediated conjunctivitis after CPT	Control	Total
TEST +	6	0	6	TEST +	20	0	20
TEST−	24	15	39	TEST−	10	15	25
Total	30	15	45	Total	30	15	45

Sensitivity: 20%				Sensitivity: 66.66%			
Specificity: 100%				Specificity: 100%			
Positive predictive value: 100%				Positive predictive value: 100%			
Negative predictive value: 38.46%				Negative predictive value: 60%			

**Table 3:** Sensitivity and specificity of Lacrytest (VKC *versus* control group).

	VKC	Control	Total
TEST +	8	0	8
TEST−	1	15	16
Total	9	15	24

Sensitivity: 88.88%			
Specificity: 100%			
Positive predictive value: 100%			
Negative predictive value: 93.75%			

## Abbreviations

CPT: Conjunctival provocation test
PAC: Perennial allergic conjunctivitis
SAC: Seasonal allergic conjunctivitis
SPT: Skin prick test
VKC: Vernal keratoconjunctivitis.
